# Reciprocity

**DOI:** 10.4269/ajtmh.19-0274

**Published:** 2019-10

**Authors:** Jesse O’Shea

**Affiliations:** Department of Internal Medicine, Yale University, New Haven, Connecticut

From the dimly lit hospital ward window, I watched as the sun fell asleep, shielded behind the endless rolling hills of Rwanda. The trees held strong; black soldiers, standing in resilient rows. The fleeting colors of dusk began to fade, and the crimson skies illuminated the hospital bed next to me.

Heather, a 64-year-old prisoner, was admitted for altered mental status. She had progressive multifocal leukoencephalopathy, a rare and often fatal complication of uncontrolled HIV. Her face was creased by suffering, weathered from a life of remorse. A medical student fervently advocated for a change to a newer, costlier, antiretroviral medication, despite knowing she may have played a part in the genocide against his family, the Tutsi people, 20 years earlier. Another prisoner, donning a cap with a red cross and a bright orange cotton outfit, acted as Heather’s caretaker.

Next to Heather lay Joseph, a 53-year-old farmer. Earlier today, he arrived at the University Teaching Hospital of Kigali, Rwanda’s largest tertiary care facility, complaining of shortness of breath—no longer able to tend to his farm animals. I too had just arrived at Kigali, but from Connecticut, with a goal of medical education. University Teaching Hospital of Kigali serves all patients, including prisoners, although the hospital tends to be more frequented by those who cannot afford care at newer private facilities. Nurses are in short supply, and family members are tasked with the daily care of patients, from bathing and feeding their loved ones to obtaining and, oftentimes, administering medications.

Rounding on Joseph this morning, 11 white coats marched toward his bed—three residents (including myself) and eight medical students. I led the pulmonary examination as the medical students helped translate. The university and Rwandan government recently expanded their capacity for medical students, quadrupling their numbers in the last 2 years. Hungry for knowledge, the students eagerly listened to Joseph’s lungs together; stethoscopes pressed against his body like being hugged by an octopus.

“Even the white man has come to help us,” Joseph’s sister said in Kinyarwandan, smiling and pointing to me. Smiling back, I told her it was nice to meet her. However, I did not know at the time that Joseph was dying. Later, he would have to be intubated for pan-resistant *Pseudomonas aeruginosa* pneumonia, a so-called superbug. He only had two previous health-care interactions in his life.

As we loomed over his bed, we assessed the pertinent findings: fevers, hypoxia, rhonchorous lung sounds, no swelling in the legs, no thrush in the mouth, and no obvious skin rashes. We hurried off to the light box to review his chest X-ray, passing by a poster on the entrance to the ward illustrating the signs and symptoms of Ebola—a reminder to always consider Ebola in our list of differential diagnoses. We talked pathophysiology as the Rwandan residents quickly showcased their fluency. An enthusiastic medical student blurted out multifocal pneumonia, noting the infiltrates were scattered throughout his lungs. I was quickly impressed by the level of discussion among the team and curious to see the patient’s treatment course and evolution. They discussed clinical trials in various countries around the world and cited guidelines from American societies, looking to me for expertise.

There is an unspoken assumption in the United States that Americans go to Rwanda “to help Rwanda.” Yet, it became clear that the team was not in need of *my* help. No, I am not this mythical American doctor who can cure all or even teach you everything. I am a part of you, I’m just like you, and I am learning *with* you. In the case of medical education, U.S. trained physicians are likely less proficient at bedside procedures, less accustomed to working with minimal or no supervision, and have limited firsthand experience with tropical diseases.

On rounds, the systemic differences from other visiting physicians were clear. “I’ve never seen a case of malaria before,” an American cardiology fellow muttered to me. “Back home, we would have gotten an magnetic resonance imaging (MRI) immediately,” he said with authority. Phrases like “this would never happen in the U.S.,” or “surgery would have been performed days ago” would be whispered out, highlighting the seemingly utopian U.S. health system and the otherness of outside systems. I worry that this line of thinking distances us from our Rwandan colleagues.

My mind flashed back to a few months ago in New Haven, when a diabetic patient demanded to see a physician after the nurse denied him a second dinner. I thought about how I have seen a patient get eight blood cultures as part of a nosocomial fever workup and a 95-year-old woman get four head computerized tomography (CT) scans in 1 month for delirium. Despite more limited access to care, there is a resourcefulness here in Rwanda that is to be admired. If the patient cannot afford labs, the residents make decisions like whether a patient needs a transfusion based on their assessment of conjunctival pallor. Bedside ultrasounds oftentimes replaced the need for advanced imaging. As the family cares for the patient, physicians perform beside lymph node biopsies at no charge. Electrocardiogram (EKG) suction cups are cleaned and reused. Patients arrive by motorcycle, ambulance, or even wheelbarrow.

Rwanda’s success is due, in part, to its leaders building modern institutions on traditional values. They breathed life back into a civic tradition of *Umuganda*, when 1 day a month, citizens, including the president, gather together to weed their fields, clean their streets, and build homes for the poorest among them. Street crime is almost unknown. The government, working closely with outside nonprofits and American institutions, is rapidly increasing the country’s capacity to care for complex medical diseases, such as leukemia.

As every sunset brings a new dawn, the Rwandans also see a better tomorrow. I am learning from their forgiveness and reconciliation as they provide compassionate care to those who had done harm to them previously. Just 20 years ago, neighbors took up arms against each other in a country-wide genocide that took the lives of nearly one million people in only 3 months. Today, the legacy is still evident, with many in wheelchairs or ravaged by HIV, yet still able to greet me with a handshake of affection. I wonder if I would have the fortitude to be able to do the same back home.

The medical wards here are dynamic with unpredictable landscapes layered with innovation and heroism from the medical staff. I heard their stories and they heard mine; I felt I became a better physician from their experience. So as I stand looking out of the window, admiring the sun’s full-blooded colors between the miles of Rwandan hills and sky, I realize that though I am helping teach these Rwandan health providers, they are also teaching me.

